# Generalist dinoflagellate endosymbionts and host genotype diversity detected from mesophotic (67-100 m depths) coral *Leptoseris*

**DOI:** 10.1186/1472-6785-9-21

**Published:** 2009-09-11

**Authors:** Yvonne L Chan, Xavier Pochon, Marla A Fisher, Daniel Wagner, Gregory T Concepcion, Samuel E Kahng, Robert J Toonen, Ruth D Gates

**Affiliations:** 1 Hawaii Institute of Marine Biology, PO Box 1346, Kaneohe, HI, 96744, USA; 2 Biology Department, University of Hawaii at Hilo, 200 West Kawili St, Hilo, HI 96720, USA; 3 Hawaii Pacific University, 41-202 Kalanianaole Highway, Waimanalo, HI 96795, USA

## Abstract

**Background:**

Mesophotic corals (light-dependent corals in the deepest half of the photic zone at depths of 30 - 150 m) provide a unique opportunity to study the limits of the interactions between corals and endosymbiotic dinoflagellates in the genus *Symbiodinium*. We sampled *Leptoseris *spp. in Hawaii via manned submersibles across a depth range of 67 - 100 m. Both the host and *Symbiodinium *communities were genotyped, using a non-coding region of the mitochondrial ND5 intron (NAD5) and the nuclear ribosomal internal transcribed spacer region 2 (ITS2), respectively.

**Results:**

Coral colonies harbored endosymbiotic communities dominated by previously identified shallow water *Symbiodinium *ITS2 types (C1_ AF333515, C1c_ AY239364, C27_ AY239379, and C1b_ AY239363) and exhibited genetic variability at mitochondrial NAD5.

**Conclusion:**

This is one of the first studies to examine genetic diversity in corals and their endosymbiotic dinoflagellates sampled at the limits of the depth and light gradients for hermatypic corals. The results reveal that these corals associate with generalist endosymbiont types commonly found in shallow water corals and implies that the composition of the *Symbiodinium *community (based on ITS2) alone is not responsible for the dominance and broad depth distribution of *Leptoseris *spp. The level of genetic diversity detected in the coral NAD5 suggests that there is undescribed taxonomic diversity in the genus *Leptoseris *from Hawaii.

## Background

Images of colonial corals illuminated by sunlight are dominant in the literature, in part because the majority of hermatypic coral species are found in the top 30 m of the photic zone [1]. This distribution reflects their reliance on sunlight to support their photosynthetic symbiotic dinoflagellates, partners that reside within the gastrodermal cells of all hermatypic corals. These dinoflagellates (genus *Symbiodinium*) enhance calcification [2] and translocate fixed carbon to the host where it is respired, and provides the majority of the metabolic needs of the host [3]. This intimate symbiosis between corals and dinoflagellates underpins the ecological success of corals in the nutrient poor environments of tropical and subtropical marine ecosystems [2].

Despite the general public perception that zooxanthellate scleractinian corals are confined to shallow well-lit waters, they exist throughout the entire euphotic zone, from full sunlight to virtual darkness [4]. Zooxanthellate corals have been recorded as deep as 165 m in the Pacific (*Leptoseris hawaiiensis*, Johnson Atoll [5]). In Hawaii, the hermatypic coral genus *Leptoseris *(Family Agariciidae) is present at shallower depths, but deep water surveys found *Leptoseris *dominates the mesophotic zone [6], where it is apparently able to photosynthesize at light levels as low as 1% of the surface light intensity [6,7]. Previous deep water surveys reported three dominant *Leptoseris *species from Hawaii, *L. hawaiiensis*, *L. yabei *(previously unknown to Hawaii), and an undescribed congeneric species, and highlighted the need for more research on these mesophotic corals, particularly their systematics [6,7].

What underlies this extraordinary physiological range and depth distribution of *Leptoseris *corals is unknown, but it is likely that attributes and adaptations in both the endosymbiotic community and coral host are involved [8]. Examining this symbiotic association at the limits of the photic zone may help us understand the interactions by which reef corals cope with and adjust to extremes in the environment. To begin to unravel the traits that allow *Leptoseris *spp. to exploit deep habitats, this study uses molecular genetic techniques to examine the interaction between endosymbiotic dinoflagellates in the genus *Symbiodinium *and mesophotic corals in the genus *Leptoseris*. Because of the difficulties inherent in collecting samples below traditional SCUBA depths, this study represents one of the first to examine the genetics of coral-algal associations at depths below 67 m in Hawaii.

Under different light regimes, coral hosts and their endosymbiotic dinoflagellates maintain high rates of photosynthetic carbon translocation by photoacclimation. This is achieved in some corals and their endosymbionts by changes in the cellular concentrations of photosynthetic pigment [9]. However, some symbiotic organisms (e.g. *Anemonia viridis *[10]) show minimal changes in the photosynthetic pigments of their endosymbionts with changes in light. These differences in physiological flexibility may reflect taxonomic differences in the composition or identity of the endosymbiotic dinoflagellate communities in the different coral species [11]. Although it was initially believed that all corals hosted a single species of dinoflagellate (*Symbiodinium microadriaticum*. Freudenthal, 1962), molecular genetics have demonstrated that the genus *Symbiodinium *is extremely diverse and a single coral can host multiple, co-occurring endosymbiotic strains [12,13]. Phylogenetic analyses of the genus *Symbiodinium*, based primarily on nuclear ribosomal genes, have led to the current recognition of eight major lineages or clades (A through H) [12,14] that are each partitioned further into sub-cladal types, most frequently using the quickly evolving internal transcribed spacer regions of the ribosomal DNA operon [15].

To date, several patterns have been detected in the distribution of *Symbiodinium *taxa with depth (all studies ≤ 40 m), suggesting that light tolerance in *Symbiodinium *may drive the distributions of host coral colonies [16-18]. Irradiance could therefore be an important determinant in the distribution of *Symbiodinium*, with different *Symbiodinium *genotypes dominating the light environments for which their photosynthetic characteristics are best adapted [11,19].

Characterizing the *Symbiodinium *communities found in mesophotic corals such as *Leptoseris *spp. is an obvious first step in exploring how these corals are able to survive and thrive at such depths. Given the unique environmental characteristics and extremely low light levels in the mesophotic zone, we hypothesized that *Leptoseris *spp. from the deep mesophotic (67-100 m) would host highly specialized and unique *Symbiodinium *types found only at these depths.

## Results

**Figure 1:**
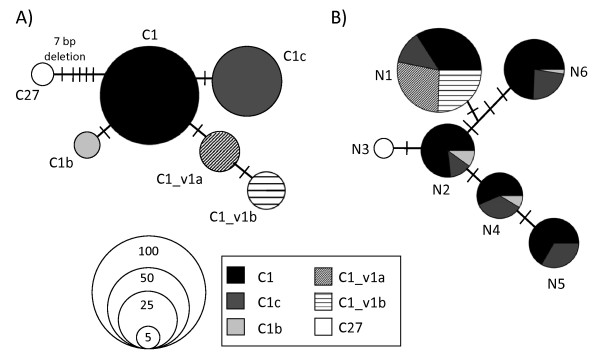
**Genotype networks showing relationships between types for A) *Symbiodinium *ITS2 types and B) *Leptoseris *mitochondrial intron NAD5**. For both A) and B) number of clones indicated by the size of the circle to scale, and hatch marks indicate base pair changes/indels between genotypes. For B) pie contents indicate the frequency of *Symbiodinium *ITS2 types recovered from the six *Leptoseris *NAD5 haplotypes.

Six *Symbiodinium *genotypes were recovered and analyzed from 15 deep (67-100 m) *Leptoseris *spp. samples. All were phylogenetically identified as belonging to clade C. Four of the sequences have been previously published (C1_ AF333515, C1c_ AY239364, C27_ AY239379, and C1b_ AY239363) and two were novel and differed from known sequences by one or two base pairs (bp). These sequences were named C1_v1a and C1_v1b, because they differed from type C1 by one and two bp, respectively. C27 includes a seven bp indel, one bp indel, and three polymorphic sites (Figure 1A).

The 15 *Leptoseris *samples grouped as six mitochondrial NAD5 haplotypes, identified by seven polymorphic sites and named N1-N6 (Figure 1B).

The association between *Symbiodinium *type and host mitochondrial haplotype was not random (Fisher's exact test, 10,000 random permutations, p < 0.01). *Leptoseris *corals with haplotypes N2, N4, N5, and N6 (n = 10 colonies in total) hosted endosymbiont communities dominated by *Symbiodinium *types C1 and C1c (Table 1). *Leptoseris *corals with haplotype N1 (n = 4 colonies) had more diverse *Symbiodinium *communities, harboring C1, C1c, C1_v1a and C1_v1b. N1 was the only coral haplotype to associate with *Symbiodinium *types C1_v1a and C1_v1b, which always co-occurred in a sample. *Leptoseris *haplotype N3 (n = 1) was the only sampled coral that hosted *Symbiodinium *type C27 (Table 1). It is important to note here that *Symbiodinium *type C1 always co-occurred with either one or a combination of the types C1c, C1b, C1_v1a and C1_v1b. Because intragenomic variation at the ITS-2 locus is high in *Symbiodinium *[20], one explanation would be that these co-occurring types represent intragenomic variants within the same symbiont genome rather than distinct genomes.

**Table 1:** Number of clone sequences of each ITS2 type, the total number of clone sequences included (In.) in the analysis, and number of clone sequences excluded (Ex.) for each *Leptoseris *spp. (n = 15) sampled showing host location, depth, and haplotype.

				**ITS2 type**						**No. of clones**	
				
**Sample Name**	**Location**	**Depth (m)**	**MtDNA**	**C1**	**C1_v1a**	**C1b**	**C1c**	**C27**	**C1_v1b**	**In**.	**Ex**.
N1_67m_1	20°47.840'N 156°43.048'W	67	N1	1	3				6	10	8

N1_70m_1	20°57.015'N 156°45.001'W	70	N1	1	6				2	9	9

N1_82m_1	20°47.675'N 156°43.047'W	82	N1	5	4				4	13	13

N1_98m_1	20°47.977'N 156°43.088'W	98	N1	9			6			15	4

N2_68m_1	20°57.015'N 156°45.001'W	68	N2	6			1			7	2

N2_68m_2	20°57.015'N 156°45.001'W	68	N2	2		3	1			6	1

N2_76m_1	20°56.504'N 156°45.418'W	76	N2	15			2			17	6

N3_74m_1	20°56.458'N 156°45.521'W	74	N3					4		4	9

N4_74m_1	20°56.458'N 156°45.521'W	74	N4	9		1	5			15	4

N4_74m_2	20°56.458'N 156°45.521'W	74	N4	4		1	3			8	1

N5_74m_1	20°56.458'N 156°45.521'W	74	N5	8			4			12	4

N5_74m_2	20°56.458'N 156°45.521'W	74	N5	10			5			15	2

N6_79m_1	20°48.821'N 156°42.640'W	79	N6	7			1			8	2

N6_82m_1	20°47.675'N 156°43.047'W	82	N6	14			6			20	5

N6_100m_1	20°47.624'N 156°43.096'W	100	N6	8		1	2			11	6

## Discussion

*Leptoseris *corals are some of the deepest-dwelling zooxanthellate corals in the world [7] and the biological attributes that underpin the ability of this genus to thrive across such a large depth range (as deep at 165 m [5]) are central to our understanding of limits of the coral-endosymbiont interaction. Intracellular photosynthetic dinoflagellate symbionts of the genus *Symbiodinium *are pivotal to the success of corals as a group and are known to be taxonomically and physiologically diverse [12]. We thus hypothesized that the *Symbiodinium *communities hosted by *Leptoseris *spp. in the mesophotic zone might be highly specialized to this environment and that this would be apparent as a pattern in the distribution of *Symbiodinium *types hosted by *Leptoseris *spp. over a depth gradient. Surprisingly, our data do not support this hypothesis; *Leptoseris *spp. sampled at 67 m and deeper, host *Symbiodinium *types commonly found in shallow-water corals across the Pacific [21]. Although endosymbiont diversity will vary by host species, this finding contradicts studies examining endosymbiont diversity in corals sampled across shallower depth gradients (≤ 40 m) that observed partitioning of *Symbiodinium *communities at the level of clade [16] and ITS2 types [17,22] by depth, and therefore, evidence for depth-based ecological function in symbionts. We found *Symbiodinium *ITS2 types C1 and C1c in mesophotic zooxanthellate corals. ITS2 type C1 has also been found in two *Leptoseris incrustans *colonies sampled in Hawaii between 10-20 m depth [21]. The generalist C1 *Symbiodinium *types are widely distributed both geographically and environmentally [21].

Solar radiation is a major determinant of photosynthesis, and therefore influences the amount of carbon translocated to the host, and the phototrophic contribution to the animal [23]. When light declines with depth, without photoacclimation, carbon fixation rates and the amount of translocated carbon declines [23]. *Leptoseris fragilis *in the Red Sea exhibit large changes in photosynthetic pigment concentrations with changes in depth [24]. Our results from *Leptoseris *spp. in Hawaii suggest that this may be a capacity of generalist *Symbiodinium *types such as C1 and C1c. However, confirming whether these abundant, generalist types have the ability to photoacclimate across the depth range under consideration here will require pigment studies and endosymbiont density counts and will be an important component of future research on the deep water corals in Hawaii.

Despite the focus on *Symbiodinium *and its ability to photoacclimate, the coral host can also influence photoacclimation. Research on *Leptoseris fragilis *in the Red Sea has shown possible photoadaptations in host light-harvesting systems that may enhance photosynthetic performance [25]. These include fluorescent pigments that may convert light at depth to wavelengths useable for photosynthesis [4], plate-like growth forms, and morphological adaptations like conical knobs that may serve as coral "light traps" [24]. *Leptoseris *in Hawaii also possess these three adaptations, but their influence on photosynthetic performance in *Leptoseris *spp. in Hawaii has yet to be directly demonstrated.

Furthermore, *Leptoseris *could differ from shallow corals in its reliance on phototrophic carbon because this coral could obtain more nutrients from feeding [26], or have reduced needs for nutrients with slower growth rates and lower metabolism [27]. For example, *L. fragilis *has trophic adaptations that may be responsible for minimizing their dependence on photosynthetically fixed carbon [28]. *L. fragilis *has a perforated gastrovascular cavity, resulting in a flow-through system where microscopic particulate organic material such as detritus, bacteria, and plankton can accumulate [28]. As light decreases with depth, greater reliance on feeding heterotrophically (rather than on phototrophy) may enable these corals to survive. However, to date, no studies have examined the relative contribution of photosynthetically fixed carbon to the daily energy budget of corals at these depths in Hawaii; such studies are critical to a more comprehensive understanding of *Leptoseris's *spp. broad depth distribution.

Given the known slow rate of evolution in coral mitochondrial DNA [29,30], the six distinct coral haplotypes we found likely represent multiple species and highlight unrecognized diversity in this coral genus. In a previous study using the NAD5 marker, Concepcion et al. (2006)[29] found no variation between species within the genus *Acropora *and *Pocillopora*, but for the genus *Porites*, *P. asteroides *and *P. compressa *differed by two indels, and *P. porites *and *P. compressa *by four single bp changes.

Interestingly, we found different *Symbiodinium *communities associated with different mitochondrial NAD5 haplotypes. Four of the coral mitochondrial types (N2, N4, N5, and N6) were dominated by C1 and C1c endosymbionts, however, N1 (n = 4 colonies) and N3 (n = 1 colony) showed different patterns of symbiont association (with C1, C1_v1a, and C1_v1b and with C27, respectively).

## Conclusion

This study was a natural first step to exploring the biological traits that allow *Leptoseris *spp. to persist and dominate at mesophotic depths (i.e. 67 to 100 m depth). We found common shallow-water *Symbiodinium *types at depths not previously recorded for these endosymbionts. We also found genetic variability at mitochondrial NAD5, which suggests undescribed taxonomic diversity in *Leptoseris*. Mesophotic coral communities are found beyond the limits of traditional SCUBA diving and as a result, their ecology is poorly understood [6,7]. An understanding of the mechanism(s) by which reef corals adjust to extremes in the environment and the limits inherent to these mechanisms provides insights into the future responses of deep and shallow reef communities to environmental change. Our study indicates that a specialist symbiont is not a prerequisite for existence at environmental extremes.

## Methods

Mesophotic corals (67-100 m depth) (n = 15) were collected using the Hawaii Undersea Research Laboratory's (HURL) manned submersibles, Pisces IV and V, during two cruises (October 2006 and December 2007) to the Au'au Channel (see Table 1 for sampling locations) between the islands of Maui and Lanai aboard *R/V Kaimikai-o-Kanaloa*. *Leptoseris *spp. fragments were broken off using the submersible's mechanical arm, brought to the surface and preserved in 95% Ethanol or DMSO buffer, or frozen at -80°C. Species level identifications of *Leptoseris *samples were problematic due to: (1) the small sizes of some of the collected specimens (due to breakage from the mechanical arm) making identification impossible, and (2) the finding of morphotypes that had previously not been reported from Hawaii [7,31]; therefore we refer to all the samples as *Leptoseris *spp. Sample size was limited due to the logistical challenges of obtaining deep-water specimens.

Genomic DNAs containing both the host and endosymbiotic dinoflagellates were extracted using a Guanidinium extraction protocol [32]. The *Symbiodinium *ITS2 rDNA marker was amplified using the primers itsD (forward; 5'-GTGAATTGCAGAACTCCGTG-3') and ITS2Rev2 (reverse; 5'-CCTCCGCTTACTTATATGCTT-3') [32,33] and *Sahara *DNA polymerase (Bioline, Randolph, MA, USA), a heat-activated high-fidelity complex of enzymes and immolase. PCRs totaled 25 μL and consisted of 2.5 μL of 10× PCR Buffer (Bioline), 0.5 μL of each primer (10 mM), 0.5 μL (2.5 mM of each dATP, dCTP, dGTP, and dTTP), 0.1 μL of DNA polymerase, 1.0 μL of DNA, and the remainder of 25 μL water. The ITS2 region was amplified with an initial denaturation step of 95°C for 10 min, followed by 35 cycles at 94°C for 30 s, 52°C for 30 s, and 72°C for one min, followed by a final extension step of 72°C for 10 min. Amplified products were cloned using the *CloneJET *PCR cloning kit (Fermentas, Glen Burnie, MD) with the pJET1.2/blunt plasmid and β-select gold efficiency cells (Bioline). Colonies were picked and initially screened for inserts of the correct size with the pJET 1.2 Forward and Reverse sequencing primers. PCR screens of the correct size were treated with exonuclease I and shrimp alkaline phosphatase [34] and sequenced at the University of Hawaii's Advanced Studies in Genomics, Proteomics, and Bioinformatics Facility.

To explore the potential association between coral and endosymbiont genotypes, the corals were genotyped using the NAD5 5' intron. Coral host DNA of the NAD5 5' mitochondrial intron was amplified and sequenced using protocols described in [29] and the primers NAD5_700F (5'-YTGCCGGATGCYATGGAG-3') and NAD1_157R (5'-VCCATCYGCAAAAGGCTG-3') [29].

Chromatograms of DNA sequences were inspected using Sequencher version 4.7 (Gene Codes Corporation, Ann Arbor, MI, USA) and sequences were individually identified via the Basic Local Alignment Search Tool (BLAST) in GenBank. *Symbiodinium *sequences were manually aligned with the BioEdit version 5.0.9 sequence alignment software [35] and phylogenetically analyzed using statistical parsimony implemented in the program TCS version 1.21 [36]. Networks were delineated with 95% certainty, with gaps treated as a fifth state.

One potential problem associated with PCR based techniques is the overestimation of sequence diversity associated with the characterization of unique sequence types that reflect PCR error and/or intragenomic variation [37,38]. To address this and provide as conservative an estimate of biodiversity as possible, sequences included in the downstream analysis were screened for the following criteria: (1) sequences had either been published previously and the sequences retrieved and verified in multiple independent studies, or (2) were recovered in this study three or more times from clone libraries representing three or more independent coral samples. In addition, ITS2 folding was checked using previously published *Symbiodinium *ITS2 structures as templates [38,39] in the ITS2 database [40,41] and manually edited using the software 4SALE [42,43]. Potential pseudogenes were identified by significant changes to the 5.8S sequence not observed in *Symbiodinium *or in other closely related dinoflagellates [38] and changes to the secondary structure of the ITS2 RNA molecule likely to disrupt the functional fold.

A total of 246 sequences identified as *Symbiodinium *were recovered from 15 deep (67-100 m) *Leptoseris *spp. samples. Of the 68 unique sequence types, 55 were recovered only once and six were recovered twice; these 61 sequence types were dropped from the analysis. The secondary structure and folding analysis of the remaining seven sequence types identified a potential pseudogene that appeared non-functional (data not shown) and this was also dropped from the analysis. The number of excluded and included sequences are shown in Table 1. The six sequences retained for downstream analyses comprised 170 of the original 246 clones sequenced (69%).

All new sequences were submitted to GenBank and can be found under accession numbers FJ919240-FJ919245 for *Symbiodinium *and FJ919234-FJ919239 for *Leptoseris*.

## Authors' contributions

YC obtained funding for the molecular analysis, collected the molecular data (including the DNA extraction, PCR amplification, and cloning of *Leptoseris *and *Symbiodinium*), conducted the data analyses, and drafted the manuscript. XP helped with the molecular data collection and contributed extensively to the manuscript. MF obtained funding, helped with the molecular data collection, and edited the manuscript. DW collected the samples and contributed to the manuscript. GC contributed to the molecular data collection and edits to the manuscript. SK collected samples, obtained funding for sample collection, and commented on the manuscript. RT designed the study and helped revise the manuscript. RG designed the study and contributed extensively to the manuscript. All authors read and approved the final manuscript.
